# The Psychological Motivations to Social Innovation and Transmitting Role of Social Worth

**DOI:** 10.3389/fpsyg.2022.850783

**Published:** 2022-03-28

**Authors:** Mei-Lan Lin, Tai-Kuei Yu, Andi Muhammad Sadat

**Affiliations:** ^1^Department of Hospitality Management, Southern Taiwan University of Science and Technology, Tainan, Taiwan; ^2^Department of Business Administration, National Quemoy University, Kinmen, Taiwan; ^3^Department of Management, Faculty of Economics, Jakarta State University, Jakarta, Indonesia

**Keywords:** social innovation, idealism, ecological concern, prior experience, perceived social worth, pro-social motivation, perspective taking

## Abstract

Social innovation has a great chance to overcome problems in complex environments. Individuals’ concern for environmental, social, and ethical issues has gradually grown, prompting the rise of new types of consumers, who shift their environmental concerns into action. Social entrepreneurship participants mostly act as beneficiaries and initiators in the process of social innovation. Social exchange theory explains the linkage between individual psychological factors and personal social cognitive perceptions that inspire social innovation intention. The current research framework is constructed to inspect the individual mental process of psychological motivation associated with social innovation intention. The purpose is to understand the relationships between the psychological level of moral idealism, ecological concern, and prior experience on cognitive perceptions of social worth; subsequently, social worth, prosocial motivation, perspective-taking, and positive feelings are examined to discover their influence on social innovation behavioral intention. The transmitting role of social worth exercises a transformative function between participants’ psychological motivation, social cognition, and social innovation intention. The research is conducted using partial least squares (PLS) analysis software. The research results reinforce our understanding of theories of individual psychological motivations on social innovation. The findings also offer some suggestions for sustainability education to social enterprise practitioners with respect to recruiting young people and continuing to generate new ideas.

## Introduction

Social innovation is a very important way to reduce or avoid environmental damage. Consumers around the world want to buy more environmentally friendly products or services (Albort-Morant et al., 2016). Individuals’ concern for environmental, social, and ethical issues has gradually grown, prompting the rise of new types of consumers, who have shifted their environmental concerns to include social innovation actions (Palacios-González and Chamorro-Mera, 2018). However, the income framework is not the primary basis for social entrepreneurs who seek to strengthen social change and innovation: they are more concerned with results and social changes.

Social innovation has a great opportunity to overcome problems in internet environment, particularly when conventional problem solving is ineffective (Antadze and Westley, 2012). The community has a desire to continuously create, adopt, and diffuse innovations that can result in prosperity for people who are involved in a variety of business and economics innovations (Pol and Ville, 2009). It clearly shows that innovation is absolutely necessary to support human life, including social innovation, which is expected to improve the quality of human life and to get effective network through social media and digital world (Luczak, 2018; Torres and Augusto, 2020).

Furthermore, Heiskala (2007) defines social innovation as resources that can strengthen the well-being of a certain group and improve its economic and social performance. The concept of social innovation is systematized to make society better. Slimane and Lamine (2017) believed that social innovation is a specific form that must be novel and original, and its purpose is to improve social welfare and generate social value (Hechavarría and Welter, 2015). Participants in the process of social innovation are mainly its beneficiaries and initiators. At the individual level, the educational model also has a certain degree of influence on developing personal psychological concepts. Concepts of social innovation learned from activities in relevant courses include cognitive perception experience and knowledge, as well as intention motives. However, even when education is able to cultivate and motivate individuals to make the world better, the social innovation process remains complicated.

Palacios-González and Chamorro-Mera (2018) pointed out that the effectiveness of individual perceptions of capability can increase the potential for behavioral intention. This refers to the individual recognition that a particular behavior can influence the environment or solve a problem. The current study posits that the effects of social cognitive action can encourage individuals to perform better and solve social and environmental problems. Then, the social innovation process intention is influenced by the actors’ perceptions of the impacts caused by these social activities: some studies show a strong correlation between perceived value and intention.

In this study, two antecedents that may influence social innovation intention are proposed: prosocial motivation and perspective taking; furthermore, social worth plays a role of transmitting psychological motivations to social cognitive perception. Malti et al. (2009) stated that engaging in prosocial activities provides opportunities to show concern for others. Since social innovation shows one’s concern for positive activities that have a broad impact, individuals may aggressively seek out ways to fulfill social innovation intention. Social action often generates positive feedback that strengthens one’s motivation to act more (Crocetti et al., 2016). Thus, there is a reciprocal relationship between prosocial behavior and perspective taking, which can affect a person’s intention to perform a specific behavior. Individuals with a perspective taking mindset tend to spend more time considering critical stakeholders’ problems.

Most of the literature available in the field of entrepreneurial intention or more specifically social entrepreneurship came from Europe and other Western countries. Environmental factors affecting the process of social innovation are very different across countries (Tiwari et al., 2017). Previous research has mostly used organizational theory and system theory to discuss the influence of social innovation on society and enterprises: the individual level has rarely been the focal point. Moreover, social entrepreneurs’ individual characteristics and abilities are the major topics in social entrepreneurship research. The current study seeks to better understand the personal psychological motivations and social cognitive perceptions that explain individual behavioral intentions for social innovation with Asia data. Social worth is also empirically examined by transmitting several antecedents of psychological factors, prior experience, idealism, and ecological concern into cognitive perception. Then, the social cognition of prosocial motivation, perspective taking, and positive feelings are surveyed and scrutinized in terms of their influences on social innovation intention and social worth. Social worth generates direct and indirect effects and transmits individual psychological and social cognitive motivations on social innovation intention. The social innovation literature mainly focuses on qualitative interviews or theoretical constructions, and seldom uses quantitative surveys. Our research results can strengthen social innovation knowledge in terms of theory and practice, and thereby inspire students to solve social problems and innovate.

## Literature Review

In this study, factors of ecological concern, idealism, and prior experience were affective and psychological. From an affective perspective, the psychological factors included the ability to react emotionally to the suffering of others; those with self-oriented motives interpreted the nature of empathy in social entrepreneurship and transmitted social worth as feeling capable and valued.

### Ecological Concern and Perceived Social Worth

When personal perceptions have a high degree of social influence, individuals will have a high degree of social concern, and they may have a higher willingness to perform social innovation. Concern is a measurable cognitive attitude—it refers to a personal thought or belief regarding a specific issue, as well as an analysis of the severity of possible social outcomes. In the existing literature, it was consistently found that individual ecological concern and socially responsible behaviors had a positive relationship. For example, Ellen (1994), Roberts (1996), Straughan and Roberts (1999), and Akehurst et al. (2012) pointed out the impacts of ecological concern and individual behavior; De Pelsmacker et al. (2005) and De Pelsmacker and Janssens (2007) asserted the impact on ethical behavior (Palacios-González and Chamorro-Mera, 2018). Ecological concern refers to feelings about green issues, and is conceptualized as a single dimension in terms of the degree people care for the environment, from low to high. Ecological concern is generally defined as the awareness of environmental problems and personal willingness to become part of the problem-solving process. From the value-oriented perspective, the altruistic behavior of environmental concerns for self-interest includes investigating ways to reduce environmental threats. It is ecologically centered and there is a positive correlation between ecological concern and environmentally friendly behavior. The individual’s ecological concern comes from the fundamental belief or value of the self, which influences subsequent attitudes and ultimately leads to specific behaviors. When an individual’s environmental awareness increases, their attitude toward eco-friendliness will be positive, and they will be as environmentally friendly as possible (Trivedi et al., 2018).

Complex systems tend to remain in a state of balance: when conditions change, the system will continue to move to find a new equilibrium (Walker et al., 2004). Consumers’ value perceptions have changed—they are aware of the importance of sustainably maintaining the natural environment, and are motivated to keep the earth from being damaged quickly. Therefore, many are willing to adopt sustainable consumption behaviors by making lifestyle compromises even though this involves additional costs (Marde and Verite-Masserot, 2018; Prendergast and Tsang, 2019). Beaumont et al. (2019) mentioned that increasing attention to ecological life has been shown to affect social worth, human health, and social well-being, particularly in relation to fisheries, heritage, and recreation. Also, Bennett et al. (2019) found that ecological effectiveness has a significant effect on conservation initiatives. In turn, the following is hypothesized:

*H1*: Ecological concern is positively related to perceived social worth.

### Idealism and Perceived Social Worth

Forsyth (1992) argued that idealism refers to the degree to which a person’s actions are carried out based on the consideration of how they will affect others. This means that an idealist will avoid all forms of action that can harm others. This is in line with Li et al. (2019), who stated that the mindset of an idealist places values and principles above practical concerns. Compared with those with a pragmatic mindset, idealists are more likely to engage in charitable behavior because they are more intrinsically motivated in their charity decision making. Intrinsic motivation to be involved in charitable activity is highly associated with positive feelings, helping people, and satisfaction (Ryan and Deci, 2000).

Grant (2008) stated that intrinsic motivation is a stronger predictor of charitable behavior than extrinsic motivation, such as money or symbolic rewards (Kivetz and Tyler, 2007). Intrinsic motivation, which is the driving force for an idealist, can positively impact society through positive activities that are useful for society (Li et al., 2019). For example, Ham et al. (2019) found idealism to be a significant influential factor on student intention to implement corporate social responsibility in the future. Also, Rosnan et al. (2013) demonstrated that ethical idealism has a significantly positive relationship with corporate social responsibility attitudes. Based on the above, it can be hypothesized that:

*H2*: Idealism is positively related to perceived social worth.

### Prior Experience and Perceived Social Worth

According to O’Sullivan and Spangler (1998), the experience felt by an individual involved in an activity is related to three things: inner needs, positive emotions, and encouraging physical involvement. Meanwhile, Stamboulis and Skayannis (2003) noted that some elements of experience can be related to research objects in the tourism sector. Personal experience can be linked to visiting, seeing, learning, enjoying, or living experiences in different settings. Involvement in social activities is also usually different for each person because feelings and absorption capability depend on individual experience. For example, in a blood donor activity, Nilsson Sojka and Sojka (2003) found that the effects of long-lasting positive blood donor experiences provide critical information to the recruitment of new blood donors. Also, some findings have been discussed in several contexts, such as economics (Pine and Gilmore, 1998), tourism (Lee et al., 2011a), and shipping (Hosany and Witham, 2010). Moreover, Song et al. (2015) found there is positive relationship between prior experiences and perceived values.

Prior experience is often included in marketing research focused on social worth. Smilansky (2009) found that experiential elements can change consumers’ brand attitudes and emotions. Affective experience is also a predictor of perceived social and transforming behavior (Iglesias et al., 2011). A pleasant experience with a particular activity will increase preferences and trust in particular issues—for example, a positive and holistic experience with a brand contributes to building trustworthy relationships between customers and the brand (Brakus et al., 2009). The study of Lee et al. (2011b) had examined the significant correlation between activity experiences and prior intention. This is an example of positive experience may generate individual perceived inner value. Therefore, we postulate that there is also a meaningful relationship between prior experience and perceived social worth:

*H3*: Prior experience is positively related to perceived social worth.

### Social Innovation Intention

Social entrepreneurs are widely seen as creative innovators to immediate social problems (Baierl et al., 2014) Innovation activities are very important in encouraging contributions to society, for instance, innovators can ease the social burden of society from crisis (Bapuji et al., 2020) As explained in the expectancy (Vroom, 1964) and goal (Wright, 2007) theories of motivation, the results of individual work are a combination of expectations and goals. Milbergs and Vonortas (2004) asserted that innovation is a sophisticated, multidimensional activity in which its effects cannot be measured directly or with just one variable. In this study, social worth, prosocial motivation, and perspective taking were cognitive, and other-oriented, and were used to examine their influence in social innovation intention. Many studies had been posited the entrepreneurial intentions is a robust predictor and the strongest available indicators of entrepreneurial behavior (Lee et al., 2011a; Hockerts, 2015; Tiwari et al., 2017; Bacq and Alt, 2018). Baierl et al. (2014) also explained the intention is the most important prerequisite to subsequent behavior based on theory of planned behavior and model of entrepreneurial event. Mair and Noboa (2006) posited that the intentions are reliable and effective predictors of actual behavior and first proposed some antecedents and intentions in social entrepreneurial context. Therefore, social innovation intention could be used to observed individuals’ orientation and behavior in the future.

Kazeminia et al. (2016) indicated that individuals are interested in environmentally friendly consumption based on the interplay between affective and cognitive evaluation. As suggested by social exchange theory (Cropanzano et al., 2017), in a social relationship, when someone provides evidence of good intentions to another party, this will inspire a sense of obligation to repay the good deeds. In other words, a person is more likely to be motivated to perform a good job when they feel valued by the beneficiaries (Castanheira, 2016). Social exchange theory assists researchers by employing psychological and sociological perspectives as a lens to view social change through the parties who exchange resources (Ap, 1992).

Heiskala (2007) was helpful in defining social innovation, which emphasizes changes in societal cultures, norms, regulations, or class structures to increase collective resources and improve economic and social performance. This is in line with Dawson and Daniel (2010), who argued that broadly speaking, social innovation can be described as an effort to develop new concepts, strategies, or tools to support particular groups in achieving better well-being. Further, Westley and Antadze (2010) emphasized that successful social innovation is an enduring, complicated process; it includes the introduction of a broad spectrum of new products, processes, or programs that will fundamentally change our underlying assumptions. As mentioned by Hamburg and Bucksch (2017), the rapid growth of digital technology play an essential role in building innovations that have a significant social effect. Therefore, it is very important to continue encouraging a culture of innovation, in addition to educational institutions as well as in the entire community of students and educators in supporting positive social, economic, and technological change.

### Perspective-Taking and Social Innovation Intention

Perspective-taking is a social cognitive ability that involves seeing the world from another person’s point of view or imagining oneself in someone else’s position (Galinsky et al., 2005) and is related to positive social outcomes (Underwood and Moore, 1982). Individuals who have a high tendency of perspective-taking tend to be aware of others’ needs. Therefore, these people can be expected to be better at finding ways to help others as compared with low perspective-taking individuals (Eisenberg et al., 2015).

Perspective-taking will increase a positive attitude toward other parties due to the establishment of connections and perceived resemblance (Gamer, 2005; Goldstein and Cialdini, 2007). When individual believes that he has a similar perception about social impact activities as the target, then he will be embedded in that, such that perspective-taking has a positive influence on perceived social innovation. In the literature, there are many potential benefits associated with perspective-taking where the parties involved benefit equally (Goldstein et al., 2014). Individuals with a perspective-taker mindset tend to have greater empathy for social targets. This is a psychological amalgamation of unified thoughts or perceptions, because perspective-takers try to see the world through others’ perspectives (Goldstein and Cialdini, 2007; Ames et al., 2008). In general, the empathy construct is affected by age and is multi-dimensional (Decety and Jackson, 2004). For instance, cognitive empathy or high perspective-taking encourages a tendency to be aware of others’ needs. Furthermore, perspective-taking often leads to a number of outcomes including facilitating social interactions and interpersonal exchanges (Galinsky et al., 2005, 2008), and the intention to perform social innovation behavior (Van der Graaff et al., 2018). Following these arguments, we hypothesize that:

*H4*: Perspective taking is positively related to social innovation intention.

### Perceived Social Worth and Intention of Social Innovation

An earlier study conducted by Grant (2008) demonstrated the connection between social worth and perceived social impact: the dynamic relationship between them is an essential indicator of how a person perceives an exchange relationship (Castanheira, 2016). Perceived social worth concerns a person’s perception that their environment respects his/her actions. In this context, there is a robust connection that an action taken will receive a particular response from the surrounding environment, so it is reasonable to assume that they will be more motivated to perform a task well when they consider the potential positive effect on the beneficiary’s life. Hakanen and Roodt (2010) explained that the interaction between someone who provides certain services that have a positive impact. Finally, these individuals will also be appreciated by society, and this becomes a means for that person to fulfill their need for self-esteem and approval. As such, it can be argued that the feeling of social worth experienced by individuals who have a positive activity experience leads them to have a greater intention to perform social innovation.

A study conducted by Grant and Sonnentag (2010) on public sanitation workers showed that the perceived social worth of the work performed could reduce perceived emotional exhaustion and also improve their performance. This means that the social worth felt by a worker who performed a particular job can encourage him/her to perform better. Manifestations of pro-social behavior enable the individuals involved to obtain non-material benefits directly for their genuine care, such as gaining social recognition as social worth. In turn, we hypothesize that:

*H5*: Social worth is positively related to perceived social impact.

### Pro-social Motivation and Social Innovation Intention

Prosocial motivation is the desire to protect and promote the well-being of others (Grant and Berg, 2011). Bénabou and Tirole (2006) explained the reasons behind pro-social actions. There are combination motives that represent prosocial behavior: extrinsic, intrinsic, and reputational. Extrinsic motivation refers to an individual external reward, typically pecuniary or other material benefits. Intrinsic motivation refers to a situation in which a person takes action not because of external incentives, but due to the positive internal feelings doing so results in. Reputational motivation refers to utilizing altruistic activities as instruments to enhance a certain reputation through repetitive interactions. However, according to Comte (2015), prosocial motivation is an egoistic activity when the main objective is to gain individual welfare, but altruistic when the final objective is to improve other’s welfare. In the context of public service, employee motivation may depend on the degree to which they feel that their work could contribute to satisfying the desire to serve others (Bright, 2007; Taylor, 2008; Vandenabeele, 2009). Moreover, Pandey and Stazyk (2008) stated that individuals with a high desire to serve others have an opportunity to provide high service performance. Moynihan and Pandey (2010) found that someone who feels like they play an important role in providing services to customers is more motivated to adopt new techniques and ways to improve their service. This is in line with Moynihan et al. (2012), who found that individuals who are motivated by their work are more likely to succeed in mastering the tools used to achieve success.

Within the social entrepreneurship context, there has been an increasing motivation in moral cognition about prosocial activity, and empirical research on social entrepreneurship has found that feelings of empathy, learning experiences, and social interest exposure can benefit both actors and recipients (Hockerts, 2017). A person feels called to do something that impacts others: the focus is on social entrepreneurial prosocial motivations that are geared toward creating social change or overcoming social problems. Prosocial motivation and social goals are essential for social entrepreneurship (Dacin et al., 2010). Tiwari et al. (2022) studied 750 social start-up entrepreneurs in India; their results show a positive relationship between prosocial motivation and social innovation intention, and confirm that the role of prosocial motivation can encourage a person to commit to social innovation. Based on the above, we hypothesize that:

*H6*: Pro-social motivation is positively related to social innovation intention.

### Positive Feelings and Social Innovation Intention

When individuals judge an objective as having high and positive benefits, they reveal an explicit preference for those activities. For example, visitors’ feelings about a sightseeing location are also indicators of their cognitive evaluation of the place and their intention to visit (Kazeminia et al., 2016). Previously, Wins and Zwergel (2016) showed that the effect of cognitive action can influence the behavioral intention of individuals in terms of social responsibility. However, when individuals have a positive or negative assessment of their perceptions of social worth, it may affect the nature of their social impact activities. Negative social impact assessments have an eventual detrimental effect on the willingness to participate in social innovation activities, while positive social impact awareness can enhance it. Bacq and Alt (2018) argued that individuals with positive and satisfied emotions are more likely to enter helping situations. Positive feelings have been extensively studied with respect to learning activities and learning achievement. Vygotsky (1994) indicated that knowledge, evolution, and emotional factors cannot be separated from learning. Individual positive feelings, such as curiosity, excitement, joy, and enjoyment, are effective in increasing learning participation and continuation, while negative emotions, such as boredom and anxiety, do the opposite (Ainley and Ainley, 2011; Linnenbrink-Garcia et al., 2011; D’Mello, 2013). Individuals subjectively believe that well-controlled learning activities and the value perceived from them can stimulate their positive feelings and curiosity, and also reduce anxiety and negative emotions. Emotional state often plays a critical role in individual course achievement and satisfaction (Cho and Heron, 2015; Butz et al., 2016). Finally, Manika et al. (2021) indicated positive feelings enhance the impacts of pro-environmental technology adoption and affect subsequent behavior. Therefore, the following hypothesis is proposed:

*H7*: Positive feelings are positively related to social innovation intention.

Social objectives and impacts are the main drivers behind discussion and research on what is meant by social innovation and how it ought to be characterized (Mulgan, 2010). Therefore, a concept must be developed by doing things that meet social needs, as well as increasing public and academic awareness. In research and discussion on social innovation, efforts are often made to place the social dimension first. Social innovation must occur through the application of new ideas to solve social challenges by meeting social objectives and improving people’s welfare.

## Materials and Methods

**Figure 1:**
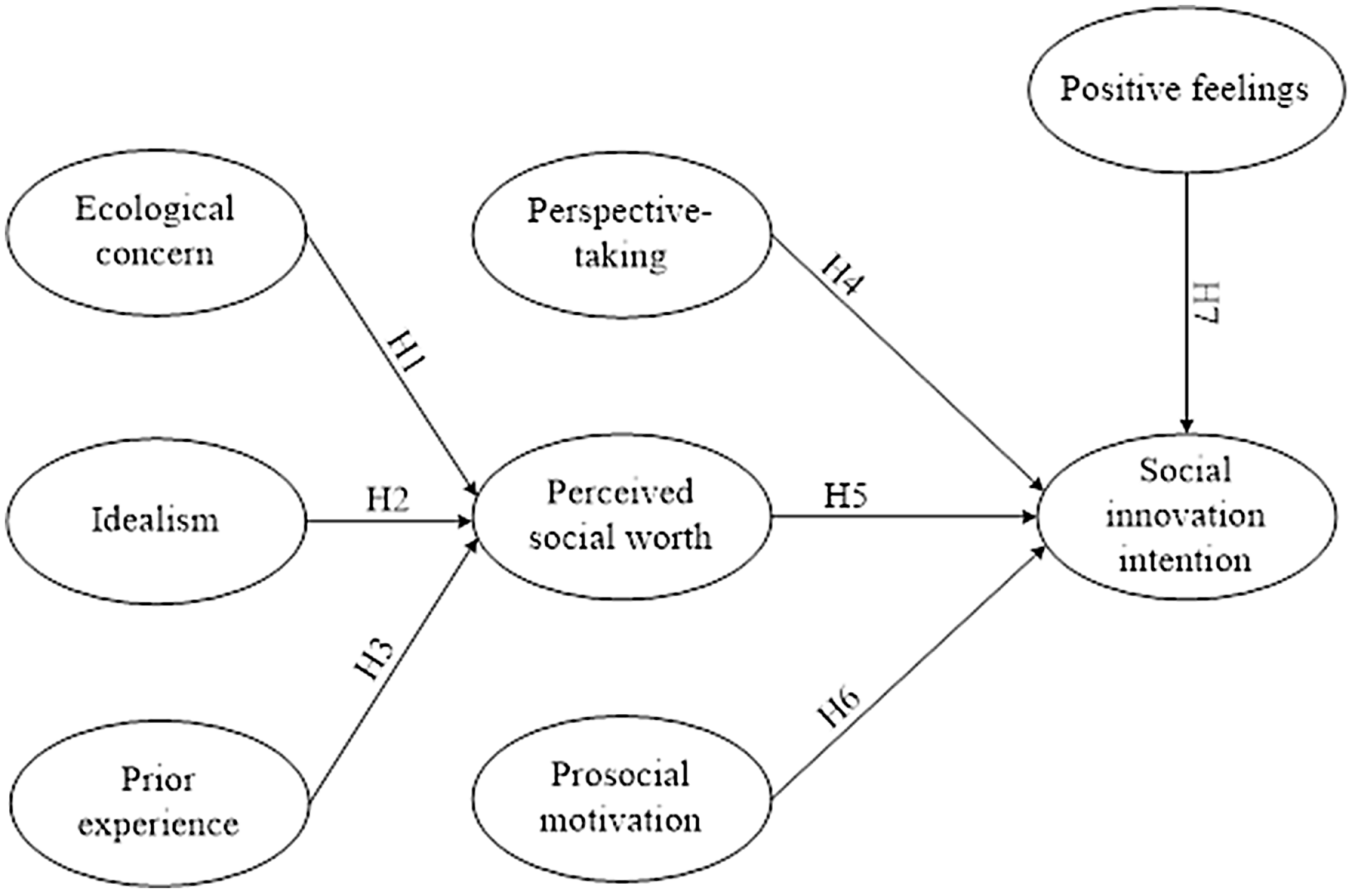
Research Framework.

The relationships between individual idealism, previous activity experience, and ecological concerns on perceived social worth were examined directly and indirectly to determine their effect on social innovation willingness. The research framework was as shown in Figure 1.

### Data Collection and Sample

Appropriate statistical methods were used to examine the effect of antecedent variables on social innovation intention based on the literature reviews. Behavioral intention is a difficult construct to evaluate, while previous studies have used elective courses to observe students’ behavioral intentions. Hockerts (2017) suggested that questionnaire respondents should consist of students taking related courses, as this can better explain individual attitudes toward a certain professional field. The study sample comprises university students in Taiwan enrolled in business courses and with volunteer experience. There are some reasons for the sample of university business students. Business schools have wide range of social entrepreneurship and innovation courses in recent years (Hockerts, 2017; Tiwari et al., 2017). Wu et al. (2022) also indicated that students engaged in business-related studies are more likely to have entrepreneurial and innovative intentions (Hmieleski and Lerner, 2016). Besides, to strength the variety of empirical study of social entrepreneurship and social innovation, data from business-oriented groups in Taiwan can represent the sample of East-South Asian. Due to the trend of global environmentalism, Taiwan’s government, industries, and organizations have begun to stress much emphasis on environmental issues for the sustainable development of Taiwan (Lin and Ho, 2008). Taiwan’s Education Bureau has also launched a program of university social responsibility to help universities and local government to adopt innovations to meet the environmental, social and sustainable compliance.

Before the formal survey, pre-test data were surveyed through the questionnaire to the 90 students of one of the private universities of Taiwan. The data were collected by purposive sampling technique under non-probability and the surveys were hand-delivered to the participants. Each participant was given approximately 20 min to complete the questions and voluntary. They were informed the survey data would be used only for academic research to ensure confidentiality and privacy of their response. Data collection took place from January to May 2020. Around 73 questionnaires were discarded due to incomplete questionnaires and those having the same answers for all questions. Table 1 summarizes the respondents’ profiles which include gender, university year, academic college, and courses enrolled related to social enterprise. Among the respondents, about 63.2% were females and 36.8% were males. The largest group in the survey sample was the second-year students in university at 53.2%, and the majority of the respondents (74.3%) was business school students.

**Table 1:** Profiles of participants (*N* = 440).

Demographics/Level	*N*	Percentage
Gender		
Male	162	36.8
Female	278	63.2
Grade		
1st Grade	73	16.6
2nd Grade	234	53.2
3rd Grade	107	24.3
4th Grade	22	5.0
Master	2	0.5
Blank	2	0.5
College		
Engineering, information and computer science	7	1.6
Business and management	327	74.3
Humanities	47	10.7
Others	55	12.5
Blank	4	0.9
Courses enrolled related to social enterprise		
Zero	82	18.6
One	147	33.4
Two	134	30.5
Three	42	9.5
Four	24	5.5
Blank	11	2.5

### Measures

The scales used in this study to measure the different model variables were well-established and adapted from the literature. To measure ecological concern, four items were adapted from Kazeminia et al. (2016). The idealism scale, comprised of four items, was adapted from Leonidou et al. (2013). Prior experience and social innovation intention were measured using Hockerts (2015) three item scales. To measure perceived social worth, six items from Bacq and Alt (2018) were used. The prosocial motivation and perspective-taking scales, comprised of five items each, were adapted from Grant and Berry (2011). The eight items of the joviality were used to assess positive feelings from Baierl et al. (2014). The questionnaire was reviewed and revised by two professors in the fields of entrepreneurship. The professors and pre-test respondents provided feedback on the clarity and flow of survey instructions. After minor modifications to individual items and formats, the final version of the questionnaire was established. In total, 38 measurement items which are revised from the previous literature. Items measuring respondents’ perceptions utilized five-point Likert-type scales anchored by 1 (strongly disagree) and 5 (strongly agree). Translation was done from English to Chinese. A pre-test and back translation was conducted to confirm the reliability of the questionnaire. The Cronbach’s alpha coefficients for all components should exceed the minimum value of 0.6 that is widely used as an indicator of reliability (Hair et al., 2011).

### Reliability and Validity

Partial least squares (PLS) were used in this study because it is a multivariate data analysis technique used to test structural equation models. The cause-effect relations between latent constructs in social science and management research are considered appropriately examined by using structural equation modeling (SEM; Hair et al., 2011). PLS-SEM estimation is more resistant to potential violations of normality than covariance-based SEM (Creixans-Tenas et al., 2020). Bootstrapping of 3,000 resamples was used to generate SEs, *t*-statistics, and confidence intervals to assess the statistical significance of the path coefficients. Following Hair et al. (2011), we selected the five most commonly used evaluation indicators, which reflected the measurement mode: (1) individual item reliability, (2) CR, (3) rho A (Dijkstra and Henseler, 2015), (4) AVE (Fornell and Larcker, 1981; Chin, 1998; Jöreskog and Sörbom, 2004; Hair et al., 2011; Bagozzi and Yi, 2012), and (5) discriminant validity. Table 2 shows the indices of the reliability and convergent validities for the scale. Table 3 depicts the discriminate validity (Heterotrait-Monotrait ratio) of the research model.

**Table 2:** Validity and reliability of research model.

	Cronbach’s Alpha	rho_A	Composite Reliability (CR)	Average Variance Extracted (AVE)
Ecological concern	0.836	0.879	0.888	0.666
Idealism	0.710	0.720	0.837	0.631
Prior experience	0.803	0.813	0.884	0.717
Perceived social worth	0.938	0.939	0.951	0.765
Perspective-taking	0.863	0.869	0.901	0.646
Positive feelings	0.941	0.942	0.951	0.709
Prosocial motivation	0.906	0.908	0.930	0.726
Social innovation intention	0.874	0.879	0.941	0.888

**Table 3:** Discriminative validity (Heterotrait-Monotrait ratio) of research model.

	Ecological concern	Idealism	Prior experience	Perceived social worth	Perspective-taking	Positive feelings	Prosocial motivation	Social innovation intention
Ecological concern								
Idealism	0.689							
Prior experience	0.297	0.416						
Perceived social worth	0.376	0.474	0.719					
Perspective-taking	0.544	0.549	0.462	0.593				
Positive feelings	0.114	0.200	0.269	0.200	0.260			
Prosocial motivation	0.435	0.526	0.639	0.717	0.652	0.299		
Social innovation intention	0.271	0.374	0.615	0.618	0.503	0.360	0.666	

With respect to individual item reliability, all factor loadings ranged from 0.754 to 0.947, which indicates good reliability, since all values exceeded 0.60 (Hair et al., 2011). However, indicator with outer loadings below 0.7 should be removed (Hair Jr et al., 2017). There were two items of factor loadings below 0.7. The AVE scores for all the constructs in this scale range from 0.754 to 0.947, which are higher than the recommended minimum value of 0.5 (Fornell and Larcker, 1981). This indicates that the scale meets the requirement of convergent validity. Cronbach’s alpha coefficients for all the constructs are greater than 0.7, and CR values are calculated for all the constructs, which ranged from 0.837 to 0.951, higher than the minimum standard of 0.7, indicating the scale meets the requirements of reliable internal consistency (Fornell and Larcker, 1981). The reliability of rho A was between 0.720 and 0.942. However, the HTMT measurement result shown in Table 3 denotes that the empirical result coefficient did not meet the 0.85 threshold required by Henseler et al. (2015). In conclusion, the overall scale’s reliability and validity meet the requirements for further research.

## The Structural Model and Results

The assessment of the structural model involves the examination of the model’s predictive capabilities and the relationships between the constructs. The criteria for assessing the structural model with PLS-SEM are mainly based on the significance of the path coefficients and the explained variance (*R*^2^; Geisser, 1974; Hair et al., 2011; Manika et al., 2021). The thresholds for explained variance (*R*^2^) are 0.75, 0.50, and 0.20 (Hair et al., 2011), while Falk and Miller (1992) indicated that the *R*^2^ should not be lower than 0.1. In this study, the calculated *R*^2^ for perceived social worth was 0.448, while for social innovation intention, *R*^2^ was 0.435.

**Figure 2:**
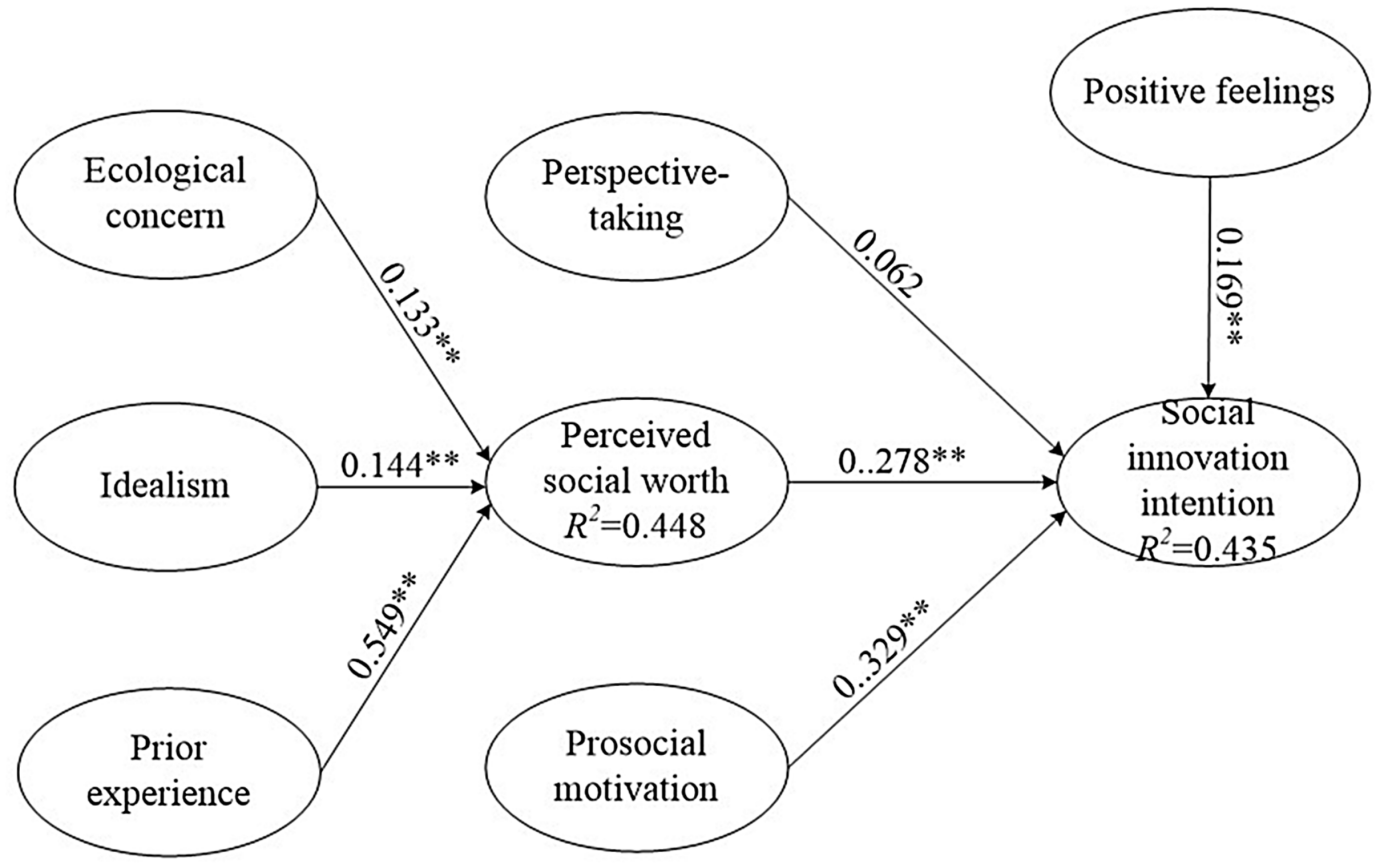
Results of the structural model testing. Value of path: standardized coefficients (β); *R*^2^: coefficient of determination. *p < 0.05; **p < 0.01.

The structural model path analysis coefficients for individual prior experience and other psychological exogenous variables into individual perceived social worth were as follows: ecological concern → perceived social worth (*β* = 0.133, *t* = 3.208); idealism → perceived social worth (*β* = 0.144, *t* = 3.036); and prior experience → perceived social worth (*β* = 0.549, *t* = 14.130). All of these reached the significance level *p* = 0.01; as such, H1–H3 were supported. The structural model path analysis coefficients for individual sociological perceptions toward social innovation intention were as follows: perceived social worth → social innovation intention (*β* = 0.278, *t* = 4.444), prosocial motivation → social innovation intention (*β* = 0.329, *t* = 5.692), and positive feelings → social innovation intention (*β* = 0.169, *t* = 4.077). As they all reached the significance level *p* < 0.01, H5–H7 were supported. However, the path of perspective-taking → social innovation intention (*β* = 0.062, *t* = 1.201) did not reach the significance level *p* < 0.05, so H4 was not supported. The model explains the variance associated with the endogenous variables: “perceived social worth” was 44.8% and “social innovation intention” was 43.5%. The PLS results for the structural model are presented in Table 4, while Figure 2 denotes the explanatory power.

**Table 4:** Estimation results for hypotheses.

	Path coefficient	*T* values	*p* values	Hypotheses
Ecological concern → Perceived social worth	0.133^**^	3.115	0.002	H1 support
Idealism → Perceived social worth	0.144^**^	3.013	0.003	H2 support
Prior experience → Perceived social worth	0.549^**^	14.003	0.000	H3 support
Perspective-taking → Social innovation intention	0.062	1.197	0.232	Not support
Perceived social worth → Social innovation intention	0.278^**^	4.448	0.000	H5 support
Prosocial motivation → Social innovation intention	0.329^**^	5.714	0.000	H6 support
Positive feelings → Social innovation intention	0.169^**^	4.085	0.000	H7 support

** *p* < 0.01.

To further investigate the indirect effects, the three individual psychological variables ecological concern, idealism, and prior experience had significant and positive indirect effects on social innovation intention. The paths of ecological concern → perceived social worth → social innovation intention has significant indirect effect (Indirect effect = 0.037, *t* = 2.474). The paths of idealism → perceived social worth → social innovation intention has significant indirect effect (Indirect effect = 0.040, *t* = 2.514). The paths of prior experience → perceived social worth → social innovation intention has significant indirect effect (Indirect effect = 0.153, *t* = 4.041). Perceived social worth also had a positive and significant direct effect on social innovation intention; therefore, these results support both a direct and indirect effect on social innovation intention, as shown in Table 5.

**Table 5:** Analysis of perceived social worth indirect effect results.

Specific paths	Indirect effect	*T* values	*p* values
Ecological concern → Perceived social worth → Social innovation intention	0.037^*^	2.474	0.013
Idealism → Perceived social worth → Social innovation intention	0.040^*^	2.514	0.012
Prior experience → Perceived social worth → Social innovation intention	0.153^**^	4.041	0.000

* *p* < 0.05;

** *p* < 0.01.

## Discussion

The purpose of this study is to examine psychological motivations and social cognitive variables to explain personal behavioral intentions. The relationships between psychological motivations idealism, ecological concern, and prior experience are considered to verify the effect on perceived social worth; the social cognitive variables of social worth, perspective taking, and prosocial motivation are tested to determine the effect on social innovation intention. Individuals with a tendency towards ecological and idealistic concern, as well as experience in this area, may transform the strength of social cognitive capabilities and perceived social worth into social innovation. The research results found that personal ecological concern, idealism in moral ideology, and previous experience have a significant, positive impact on perceived social worth, while perceived social worth and prosocial motivation have a significant, positive, direct effect on social innovation willingness. However, perspective-taking has an insignificant effect on social innovation intention.

### Theoretical Implications

An ecological awareness includes all ideas, values, and opinions about the environment as a place for living, it needs the state of knowledge for maintaining the methods, protecting, and shaping of the environment which in the end has a social meaning (Wierzbiński et al., 2021). So it can be said that ecological concerns has an essential effect on social environment as shown in this study that ecological concern has significant effect on perceived social worth. As mentioned by McIntyre and Rondeau (2011) that ecological concern addressed by supporting the local farmers in Canada to increase regional food supply and encourage the consumption of healthy food, which in the end will provide wider social benefits. Also, the attention of several parties in preparing organic products continues to increase significantly and is expected to continue to increase spending on ecological products and provide wider social benefits. In term of social economics, Spash (2011) stated that the rise of ecological economics can be explained as a relevant step toward integrating social and natural fields to go beyond the confines of mainstream economics view and underlining how economic growth is perceived as a means for improving the social condition.

Idealism is primarily concerned with the welfare of others. Idealists assume that people can always get the desired results when they take the right actions, and therefore it is always possible to avoid harming others. Individuals with a high degree of morality will absolutely abide by moral standards when making decisions. They also tend to have negative attitudes toward unethical behavior. In other words, the more idealistic an individual is the higher social worth that individual may perceive himself/herself to possess, and this will influence social innovation intention. These research results are consistent with Palihawadana et al. (2016). Someone with high service motivation is more likely to be motivated to perform a certain task. Pro-social motivation has a significant effect on the delivery impact of public services (Francois and Vlassopoulos, 2008), which is consistent with our findings.

Research results concerning prior experience in this study are similar with those obtained in many previous studies. Vining and Ebreo (1989) found that previous experience can generate a capability to help people and predict beneficial social behavior, such as participation in recycling programs. Previous community service experience has also been found to predict the impact of participating in ethics courses. Community experience can establish the identity of social entrepreneurs, and help them discover their social goals and values based on community experience (Pathak and Muralidharan, 2016). Prior knowledge of social issues can predict the attitude of social entrepreneurial intentions and perceived behavior control (Ernst, 2018). In addition, Yiu et al. (2014) pointed out that if the individual has prior experience, they are more likely to get involved in charity programs.

Prosocial motivation is understood to be a mental state where individuals emphasize benefiting others as their goal; however, perspective-taking does not have a significant effect on social innovation intention, such that H4 was not supported. Similar results were also obtained in previous studies, which suggest that the psychological process of perspective-taking limits creativity, because perspective-taking may emphasize societal obedience, which reduces individuals’ ability and motivation for multi-dimensional thinking. Goncalo and Staw (2006) found that focusing on the values of others emphasizes group collectivism. The value of obedience is the foundation of collectivism: it emphasizes the importance of meeting the expectations of others and maintaining harmony, which may encourage individuals to suppress innovative and unique ideas.

The transmitting function of social worth is empirically explained with respect to social intention inspiration based on psychological motivations and social cognitive perception. Some suggestions are provided to social businesses and those involved in social innovation in business, especially in the digital world.

### Managerial Implications

Social innovation advocates can help make society better. Social innovation not only strengthens the supply chain, but can also access social consciousness and green consumers. Although many companies have begun to realize the importance of corporate social innovation, they can support social innovators or alliances with social enterprises to help solve social problems. Corporations may choose to not establish a new social enterprise by themselves: multiple strategies can be used to develop their social innovation—for example, internal social businesses and strategic alliances with external social enterprises. The purpose of internal social innovation is to achieve strategic investment in reputation and competitiveness, as well as deep cooperation between social enterprises and external groups that can create new common capabilities and sources of income, and thereby provide sustainable solutions to the morbid state of society.

Corporate social innovation is usually a partnership between business entities and non-business entities, which emphasizes social impact. In the cyber world, corporations generate wide social innovation through the exchange of knowledge with external alliance partners. Tacit knowledge of social innovation in a specific context is needed to accumulate experience (Mirvis et al., 2016). Therefore, businesses and non-profit enterprises often resort to joint development of social innovation. In recent years, the sharing economy has flourished and has become interested in social innovation. By definition, social innovators need to incorporate social changes into their strategies and ultimate goals. Social innovation is becoming part of the sharing economy or cooperative economy—that is, open social innovation. Open social innovation is the application of inbound or outbound open innovation strategies, accompanied by innovations in organizations for social changes (Lowitt, 2013; Chesbrough and Di Minin, 2014; Zervas et al., 2017). In this context, innovation is defined as the process by which a person/organization/nation creates and transforms certain knowledge and technology into products, services, or processes that are useful to achieve a better quality of life. The digital era is currently growing by relying on knowledge networks and conditions for innovation, as well as opening up opportunities to apply open innovation in a social context by inviting all interested parties to get involved. For instance, by engaging any related entities to innovate by providing learning content-especially for students with special needs (Hamburg and Bucksch, 2017). Also, in recent research, Scheidgen et al. (2021) found that innovators are the main agents in dealing with the various consequences caused by COVID-19 through digital intermediaries and digital services.

## Conclusion

Do college students also think that social and environmental problems are personal responsibilities that everyone must consider? These research findings can contribute to the level of social innovation knowledge in education, industry, and government, and inspire students to solve social problems and innovate. Previous experience has a significant, positive, direct effect on perceived social worth, and an indirect effect on social innovation. Therefore, universities should arrange to include field experience for related activities in the curriculum to enhance students’ awareness of social impact and increase future employment in this area. Higher Education Institutions must actively encourage collaborative and systemic learning to engage directly with the community as part of social actors. The technology-based transdisciplinary approach is the main factor that can encourage social innovation (Kumari et al., 2020).

After understanding the psychological and sociological factors of the individual, establishing students’ social innovation literacy and planning our learning motivation strategies will provide an important reference to cultivate students’ social innovation literacy through the teaching mechanisms and methods used. The research results may encourage students to believe that they have the ability to lead social changes, and care for and contribute to society. Valter and Akerlind (2010) suggested that improving students’ way of thinking and acting skills like a researcher to include them in social problem. And promote these skills to accomplish their own tasks which in turn can help the community around them (Oganisjana et al., 2017).

The results can also strengthen our knowledge with respect to social innovation and help students build critical thinking and problem-solving skills. This is also important to the metaverse: in the future, relevant knowledge will be linked automatically, leading to much more rapid open social innovation. One limitation of this study is that the respondents were all university students from Taiwan. The survey can be utilized in other countries in the future to evaluate our results. Social innovation has become a global movement. If samples from Southeast Asian countries can be effectively collected and compared, differences among nations can be analyzed. Social innovation is not just the focus of attention of social enterprises and social entrepreneurs. For-profit businesses have also invested in the development of social innovation, either due to internal self-innovation or cooperation with non-profit organizations. The main purpose is to improve social well-being. For social issues, both companies and individuals must do their best. If we regard social innovation as a new type of social literacy, it can help to provide solutions to social problems and increase social harmony.

## Data Availability Statement

The raw data supporting the conclusions of this article will be made available by the authors, without undue reservation.

## Author Contributions

M-LL contributed to conception and design of the study. T-KY organized the database and performed the statistical analysis. M-LL and AS wrote the first draft of the manuscript. All authors contributed to the article and approved the submitted version.

## Funding

This study was funded by Ministry of Science and Technology, Taiwan, under grant research project number: MOST108-2511-S-218-007.

## Conflict of Interest

The authors declare that the research was conducted in the absence of any commercial or financial relationships that could be construed as a potential conflict of interest.

## Publisher’s Note

All claims expressed in this article are solely those of the authors and do not necessarily represent those of their affiliated organizations, or those of the publisher, the editors and the reviewers. Any product that may be evaluated in this article, or claim that may be made by its manufacturer, is not guaranteed or endorsed by the publisher.
